# An Improved Workflow for the Quantification of Orthohantavirus Infection Using Automated Imaging and Flow Cytometry

**DOI:** 10.3390/v16020269

**Published:** 2024-02-08

**Authors:** Laura Menke, Christian Sieben

**Affiliations:** 1 Nanoscale Infection Biology, Helmholtz Centre for Infection Research, Inhoffenstr. 7, 38124 Braunschweig, Germany; laura.menke@helmholtz-hzi.de; 2 Institute of Genetics, Technische Universität Braunschweig, Spielmannstr. 7, 38106 Braunschweig, Germany

**Keywords:** orthohantavirus, virus quantification, imaging, bunyavirales

## Abstract

Determination of the infectious titer is a central requirement when working with pathogenic viruses. The plaque or focus assay is a commonly used but labor- and time-consuming approach for determining the infectious titer of orthohantavirus samples. We have developed an optimized virus quantification approach that relies on the fluorescence-based detection of the orthohantavirus nucleocapsid protein (N) in infected cells with high sensitivity. We present the use of flow cytometry but highlight fluorescence microscopy in combination with automated data analysis as an attractive alternative to increase the information retrieved from an infection experiment. Additionally, we offer open-source software equipped with a user-friendly graphical interface, eliminating the necessity for advanced programming skills.

## 1. Introduction

Orthohantaviruses are enveloped viruses that contain three circular single-stranded RNA genome segments of negative sense, namely, the small (S-segment), medium (M-segment) and large segment (L-segment) [1,2]. Encoded on the S-segment is the nucleocapsid (N) protein [3], which is often used for virus quantification due to its early and abundant production [4,5,6]. Research on orthohantaviruses started with the discovery of the Hantaan virus (HTNV) in 1978 [7], and morphological and genetic insights on HTNV followed as soon as virus propagation and titration could be established in cell culture systems [8,9]. Here especially, the viral adaptation to Vero E6 cells was an important milestone [10]. Since then, several protocols for orthohantavirus titration in cell culture have been developed. The widespread foci assay titrates the number of infectious particles by detecting the viral N protein in infected cells [9,11,12]. The foci assay is closely related to the plaque assay where plaques of dead cells are visualized as empty spots following a general cell staining procedure (i.e., with methylene blue). Since orthohantaviruses are non-lytic, infected cells are detected via the expression of viral proteins. Specifically, a semisolid overlay medium ensures that after infection, viral spread can only occur as far as neighboring cells, which leads to a patch of infected cells that can be counted as a focus [9,11,12]. A derivative of the fluorescence-based foci quantification protocol is viral antigen detection via chemiluminescence, which potentially offers improved sensitivity [13]. One drawback of the foci assay is its labor- and time-consuming protocol, which takes 5–11 days depending on the viral strain [13]. Recently, a flow cytometry-based titration assay was established using Andes orthohantavirus (ANDV). This assay has been shown to reduce the time required for the detection of viral replication during infection [14]. The authors state that the tested foci assays can be compared with their improved protocol, in which ANDV detection was possible as early as 6 h post-infection.

Here, we provide an improved virus quantification protocol based on fluorescence imaging and flow cytometry that is compatible with other orthohantavirus strains such as Puumala virus (PUUV) and Tula virus (TULV). While earlier time points can also be detected via fluorescence microscopy, our data suggest that PUUV and TULV should best be quantified after 48 h. We provide an automated imaging workflow together with a data analysis package which allows for the routine quantification of large amounts of single-cell data (5000–10,000 cells) in a time-efficient way. An additional advantage of titration through imaging is the simultaneous extraction of structural information, eliminating the need for an extra experimental setting. This represents a significant benefit, enabling discrimination between the early and late stages of infection.

## 2. Material and Methods

### 2.1. Cells, Viruses and Reagents

Vero E6 cells were purchased from ATCC and grown in DMEM (Thermo Fisher Scientific, Waltham, MA, USA: DMEM (1x) + GlutaMAX^TM^-I, [+] 2.5 g/L D-Glucose, [-] Pyruvat) containing 10% fetal calf serum (FCS) (Merck, Darmstadt, Germany) and 1% penicillin/streptomycin (Pen/Strep) (Thermo Fisher Scientific, Waltham, MA, USA). Deionized water processed by an EMD Millipore^TM^ Milli-Q^TM^ advantage A10 ultrapure water purification system served as the basis for all buffers and solutions. All disposable cell culture materials were purchased from Sarstedt (Nümbrecht, Germany), TPP (Trasadingen, Switzerland), Thermo Fisher Scientific (Waltham, MA, USA) and Greiner bio-one (Kremsmünster, Austria). Nucleocapsid (N) proteins were detected using primary antibodies 5E11 (Abcam, Cambridge, UK; cat. ab34757) [15] or monoclonal antibody anti-TULV-1, generously provided by Jens Peter Teifke and Sven Reiche (Friedrich-Loeffler-Institut, Greifswald, Germany). We used Alexa Fluor 568 (cat. A11031) and Alexa Fluor 488 secondary antibodies (cat. A11029) (Thermo Fisher Scientific, Waltham, MA, USA).

### 2.2. Orthohantavirus Propagation

Hantaviruses were propagated on Vero E6 cells (ATCC: CCL-1586, Manassas, Virginia) under strict standard operating procedures using biosafety level 2 (BSL2) facilities and practice. For virus propagation, T75 flasks with Vero E6 cells were seeded to reach a density of 80–90% confluence after overnight incubation at 37 °C in 5% carbon dioxide (CO_2_). The cells were infected using a multiplicity of infection (MOI) of 0.01–0.1 in DMEM + 2% FCS + 1% Pen/Strep. After 1 h at 37 °C (+5% CO_2_), the inoculum was removed and the medium replaced with fresh DMEM + 2% FCS + 1% Pen/Strep. The infected cells were further incubated for 7–10 days, whereby after day 4, the supernatant could be collected on a daily basis and, later, either quantified passage per passage or pooled using concentration methods. Collected supernatants were centrifuged for 30 min at 1200–2000 g at 4 °C to remove cell debris then transferred into 50–100 kDa filtration units (Amicon) (Sartorius AG, Göttingen, Germany) and centrifuged at 4000 g until volume decreased to 1–3 mL. The retentates were mixed, resuspended and frozen at −80 °C until further use.

### 2.3. Preparation of Glass Coverslips for Microscopy 

A total of 200 mL washing solution containing 60% hydrochloric acid (HCl) and 40% ethanol (EtOH) were mixed, and 120 mL of HCl was added and heated to 37 °C. The solution was filtered through a pleated filter. Coverslips were washed carefully in a 1 L glass bottle on a roll shaker for at least 30 min. Afterwards the HCl/EtOH mix was discarded. Coverslips were washed 3 times with water and then dried on Whatman paper. Finally, the slides were sterilized at 180 °C.

### 2.4. Preparation of a Fluorophore-Conjugated Primary Antibodies

A total of 20 µL of the antibody 5E11 (Abcam, Cambridge, UK (cat. ab34757)) [15], together with 20 µL phosphate-buffered saline (PBS; filtered) (Thermo Fisher Scientific, Waltham, MA, USA), 10 µL NaHCO_3_ (pH 8–8.5; 0.5 M) and 0.5 µL dye conjugate (CF-488A Dye; Biotium, Fremont, CA, USA) (4 µg; 64 µM final; dye overload ca. 1:50) were incubated for 30 min at room temperature in the dark. Amersham NAP-5 Sephadex columns (Cytiva, Marlborough, USA) were rinsed with phosphate-buffered saline (PBS) before the sample was applied and eluted with PBS. Run through was collected drop-wise in a 96-well plate (well after well), and the dye/protein content of each well was measured using a NanoDrop (Thermo Fisher Scientific, Waltham, MA, USA). The wells with the highest concentration were pooled and measured again to obtain the final concentration (protocol is based on [16]).

### 2.5. Immunofluorescence Assay (IFA) for Flow Cytometry

A total of 100,000 Vero E6 cells were seeded in a 24-well plate (Corning Incorporated, New York, NY, USA) and incubated with DMEM + 10% FCS + 1% Pen/Strep for 24 h at 37 °C in 5% CO_2_. Dependent on the type of experiment, either an MOI of 1 was used or a fixed volume (5 µL or 50 µL). For infections, the cell layer should be 80% confluent. The used virus volume was filled to 300 µL per well with DMEM + 2% FCS + 1% Pen/Strep and incubated for 1 h at 37 °C. The cells were washed twice with PBS and further incubated for 48 h with DMEM + 2% FCS + 1% Pen/Strep. After washing with PBS, the cells were treated with Accutase (Merck, Darmstadt, Germany) for 5 min at 37 °C. The detached cells were transferred into a tube, which was filled with PBS and then centrifuged (all following centrifugation steps: 2000× *g* for 5 min at 4 °C). Near-IR fluorescent reactive dye (Thermo Fisher Scientific, Waltham, MA, USA) was used 1:1000 in PBS and added for 30 min at room temperature (RT). As a washing step, the tubes were filled with PBS and centrifuged. We used 4% paraformaldehyde (PFA) for 30 min at RT to fix the samples, followed by a dilution/washing step and centrifugation. The cells were permeabilized for 15 min using 0.5% saponin in PBS at RT, followed by a dilution/washing step and centrifugation. Antibodies were added in a buffer consisting of PBS, 2% FCS, 0.4% 0.5 M ethylenediaminetetraacetic acid (EDTA) and 0.5% saponin and incubated for 1 h at RT. After each antibody-staining step, two washing steps were performed. The samples were analyzed using a BD LSR-II flow cytometer using the software BD FACSDiva (version 9.0). Data were further processed using FlowJo_V10.9.0.

### 2.6. Immunofluorescence Assay (IFA) for Microscopy

A total of 100,000 Vero E6 cells were seeded in a 24-well plate (Corning Incorporated, New York, USA)) containing 12 mm coverslips (Epredia, Kalamazoo, MI, USA). Alternatively, 50,000 cells were seeded in a 48-well plate containing 7 mm coverslips. The cells were incubated in DMEM + 10% FCS + 1% Pen/Strep for 24 h at 37 °C in 5% CO_2_. Dependent on the type of experiment, either an MOI of 1 was used or a fixed volume (5 µL or 50 µL). For infections, the cell layer should be 80% confluent. The used virus volume was filled up to 300 µL per well with DMEM + 2% FCS + 1% Pen/Strep and incubated for 1 h at 37 °C. The cells were washed with PBS and further incubated for 48 h with DMEM + 2%FCS + 1% Pen/Strep. We used 4% PFA for 15 min at RT to fix the cells (Thermo Fisher Scientific, Waltham, MA, USA). As a quenching solution, we used 300 mM glycine (Serva, Heidelberg, Germany) in PBS and then permeabilized the cells in 0.1% Triton X-100 (Thermo Fisher Scientific, Waltham, MA, USA) in PBS for 30 min at RT. As a blocking solution, 1% bovine serum albumin (BSA) (Thermo Fisher Scientific, Waltham, MA, USA) was mixed with 0.1% Tween20 (Thermo Fisher Scientific, Waltham, MA, USA) in PBS and added for 30 min at RT. All following antibodies or dyes were diluted in the BSA-Tween-PBS-blocking solution. The first antibody, as well as the secondary antibody was incubated for 1 h at RT, followed by 20 min incubation with Phalloidin (1:400 dilution pf a 66 µM stock solution; manufacturer’s specification; Thermo Fisher Scientific, Waltham, MA, USA; cat. A22287) and 10 min of Hoechst (1 µg/µL; Thermo Fisher Scientific, Waltham, MA, USA; cat. H3570) staining. Between all steps, the samples were washed three times with PBS and once with water before the samples were mounted in ProLong TM Gold antifade reagent (Thermo Fisher Scientific, Waltham, MA, USA). The coverslips were dried for at least 24 h. As a sealant, CoverGripTM (Biotium, Fremont, CA, USA) was used and dried for at least 24 h. 

### 2.7. Fluorescence Microscopy

Fluorescence microscopy was performed on a Nikon Ti2-Eclipse microscope equipped with the spinning disk confocal module CSU-W1 (Yokogawa) as well as an LED widefield illumination light source (pE-4000, CoolLED). Images were acquired with a S Plan Fluor 20× and Plan Fluor 60× oil objective (Nikon, Minator, Tokyo, Japan), a PCOedge 4.2LT or a Zyla 4.2 sCMOS camera (Andor, Belfast, UK) and 405/488/561/638 nm laser lines (Omicron, Rodgau-Dudenhofen, Germany) controlled by NIS-Elements software (version 5.11.01). Z-stack images were acquired with a step size of 300 nm.

### 2.8. Data Processing and Statistical Analyses

Image analysis was carried out using CellProfiler (version 4.2.1) and NIS-Elements (version 5.11.01) (Nikon, Minator, Tokyo, Japan). Further post-processing was conducted using our custom software Datalyze (Supplementary Software). Further data processing steps and statistical analyses were carried out in NIS-Elements, Inkscape, Excel 2010 and Graphpad Prism 9. Graphical illustrations were created with https://app.biorender.com (accessed on 4 July 2023).

## 3. Results

### 3.1. PUUV and TULV Grow to Low Titers, Requiring Time-Consuming Quantifications

Recently, imaging-based virus quantification has become increasingly popular due to its ease of use and the additional amount of information contained in microscopy images [17,18]. Pseudotyped or recombinant viruses encoding a cytosolic fluorescent reporter further enable the quick detection of infected cells. However, some orthohantavirus strains grow to relatively low titers compared with other enveloped RNA viruses and a recombinant system is not yet available. In our hands, PUUV reached a 100-fold lower titer compared to influenza virus A/Puerto Rico/8/34 and La Crosse encephalitis virus (LACV), another bunyavirus. Compared to VSV, the titer was even 10^4^-fold lower (Supplementary Figure S1). The typical proliferation time is also considerably longer for orthohantaviruses, which typically require on average 10 days of incubation. Consequently, the small amount of virus material available is typically used up quickly, leading to a constant amplifications process, followed by quantifications of viral titers again for up to 10 days. We thus set out to optimize the orthohantavirus quantification methods to reduce the amount of infectious material and save time compared to the standard method.

### 3.2. We Present an Improved Flow Cytometry Workflow to Quantify PUUV and TULV with an Optimized Time Point and Reduced Material Requirement

**Figure 1:**
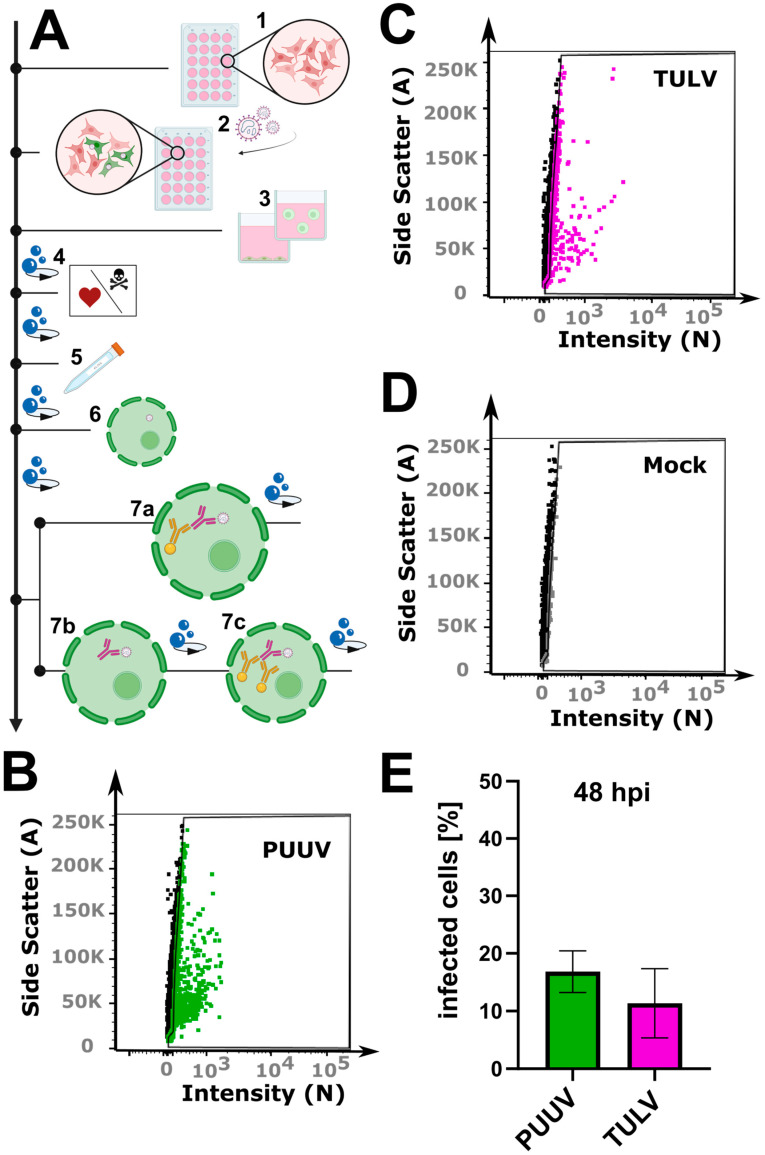
Flow cytometry workflow for quantifying orthohantavirus infection in cell culture. (**A**) Schematic overview of the protocol: (1) cell seeding; (2) infection with orthohantavirus for 1 h; (3) cell detachment with Accutase; (4) live–dead staining; (5) fixation with paraformaldehyde (PFA); (6) cell permeabilization with saponin; (7) staining with antibodies: either with a conjugated antibody (7a) or with a primary (7b) and a secondary (7c) antibody. (**B**–**D**) Exemplary gate settings for quantification of newly propagated virus; their analysis in (**E**) is a bar graph displaying the percentage of infected cells (%). Shown are mean ± SD, N = 3.

Quantifying orthohantavirus infection using flow cytometry has been described as early as 6 h post-infection for ANDV infection [14]. We thus first tested this early time point after PUUV and TULV infection using the described protocol but could not detect any infected cells (Supplementary Figure S2). Instead, 48 h post-infection enabled a robust detection of infected cells (Figure 1B–E), suggesting that the time point requires optimization depending on the virus strain used. During our optimization, we could further reduce the required cell number by 80%. While the previous protocol describes the use of a 6-well plate format (well surface area: 9.6 cm^2^), we could adjust the protocol to a 24-well plate format (well surface area: 1.9 cm^2^). For the small amount of required starting material which still allows for the measurement of 10,000 cells, we want to highlight several critical steps in our protocol (Figure 1A). First, the cells were detached from the well plate using Accutase, which we found to be superior compared to trypsin or cell scrapers, which are often used in flow cytometry assays. The cells were collected in a buffer containing EDTA to prevent the cells from sticking together. Then, a live–dead marker was included, which enables the selection of only intact cells for the final analysis. Following this staining step, the cells were fixed in 4% PFA. The subsequent antibody staining requires cell permeabilization, for which we recommend using aponin rather than detergents such as Triton X-100. Orthohantavirus infection is then detected using primary antibodies against the viral nucleocapsid protein N diluted in a buffer containing saponin to retain the cells’ permeability for the required intracellular staining. This step is followed by two washing steps and incubation with a secondary antibody, again followed by another two washing steps. Here, the use of a secondary antibody amplifies the signal since one primary antibody can bind more than one secondary antibody. However, due to the many washing steps, we observed a strong loss of cells: on average, 3–10% per washing step (Supplementary Figure S3). The use of a direct fluorophore-conjugated antibody can help overcome this limitation while retaining the use of small cell culture volume (24 well). As we describe in Section 2, the anti-N primary antibody was then conjugated to a selected bright dye in a separate process. Using a pre-conjugated primary antibody saved about 75 min during each of the quantification experiments, as well as at least two washing steps and the associated loss in cell number. As an additional benefit, the background signal was also reduced when compared to the use of a combined primary/secondary antibody in a mock infected control sample (Supplementary Figure S4). Finally, following a simple gating strategy to select only live single cells (Supplementary Figure S5), our optimized protocol enabled the counting of cells infected with PUUV and TULV after 48 h using a small number of input cells. 

Microscopy in combination with automated image analysis is a highly sensitive approach for quantifying orthohantavirus infection phenotypes. The mere counting of infected cells based on the presence of a viral protein leaves out the opportunity to use the positional and structural information of the antibody-labeled proteins within the cellular context. Hence, we wondered if we could also use an imaging-based approach to quantify infection with PUUV and TULV in cell culture. 

**Figure 2:**
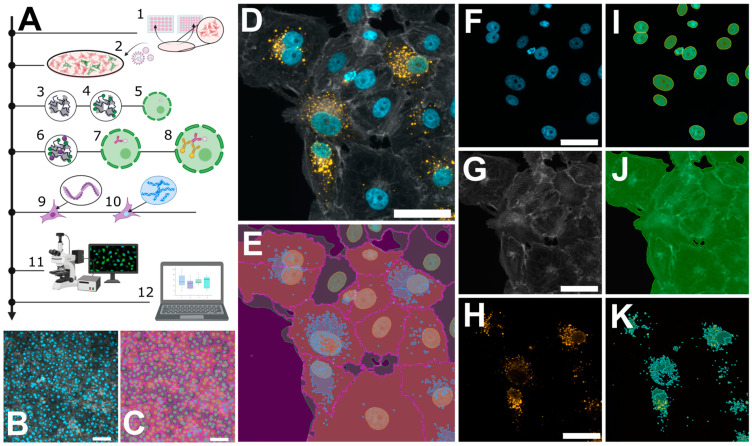
Microscopic workflow for quantifying orthohantavirus infection in cell culture. (**A**) Schematic overview of the protocol: cell seeding (1); infection with orthohantavirus for 1 h (2); cell fixation with paraformaldehyde (PFA) (3); blocking of unreacted aldehydes with glycine solution (4); permeabilization with Triton X-100 (5); blocking of unspecific binding sites using a BSA-containing buffer (6); staining with primary antibody against viral nucleocapsid protein (N) (7) and staining with a secondary antibody (8); phalloidin staining to label filamentous actin (9) and Hoechst for labelling dsDNA (10); and imaging via fluorescence microscopy (11) and analysis via custom software tools (12). (**B**,**C**) Exemplary images recorded with 20× magnification, showing ~500 cells. (**D**–**K**) Pairwise original images together with their masks resulting from software-based segmentation. (**D**) A merged image of three individual channels in (**F**–**H**): Hoechst (**F**) and its mask for nuclei recognition (**I**); phalloidin staining (**G**) for recognizing cell bodies or cell boundaries; and Anti-N staining (**H**) to reveal viral particles (**K**). The overlay of (**I**–**K**) is shown in (**E**). Scale bars = 50 µm.

**Figure 3:**
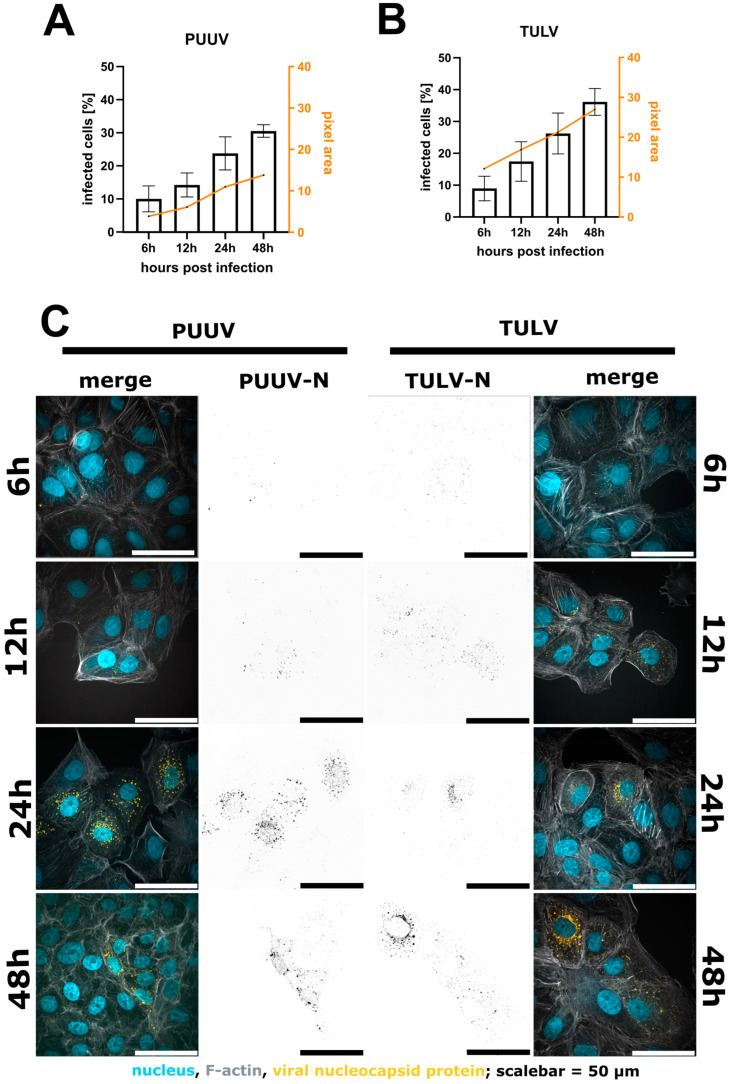
Quantification of infected Vero E6 cells 6 h, 12 h, 24 h and 48 h post-infection with either PUUV (**A**) or TULV (**B**). A comparison of the percentage of infected cells shows an increase in detected cells after 48 h compared to 6 h. We observed a correlation between the number of infected cells and the intensity per pixel. Shown are mean ± SD, n = 3. Exemplary images in (**C**). Merged image show nuclei in blue, F-actin in grey and the viral N protein in orange. Viral N is also shown in black as a separated image in the middle of the image panel.

In general, quantification via microscopy requires even lower input material due to the specific and exhaustive imaging of whole cell populations and the low loss during sample preparation. We routinely use either 12 mm coverslips in a 24-well plate or 7 mm coverslips in a 48-well plate (well surface area: 1.1 cm^2^). Compared to a 6-well format, this results in a reduction of 89% with respect to the required reagents. Vero E6 cells were infected for 48 h, as described above, then fixed with 4% PFA directly on the glass slides. No detachment and centrifugation steps were required, thus minimizing the cell loss compared to the flow cytometry workflow (Supplementary Figure S5). Nevertheless, on a 7 mm coverslip, it is still possible to acquire 15–25 non-overlapping images using a 21× objective, resulting in a cell count of 5000–10,000 cells for the evaluation, which is comparable with the flow cytometry workflow described above (Figure 2). Following fixation, the cells were permeabilized with Triton X-100 and blocked with a BSA-containing buffer, which was also used for the antibody dilutions. Since there is no risk of losing cell material during the following washing steps, we recommend using primary/secondary antibodies to increase the signal to noise. To enable single cell segmentation later on, we then stained the cell body using phalloidin as well as the nuclear DNA using Hoechst (Figure 2D–K). Images were taken at 20× magnification, whereby one image corresponds to approximately 200–500 Vero E6 cells (Figure 2B) that could still be segmented adequately as shown by the corresponding software-based mask in Figure 2C. For image processing, we used the licensed program NIS-Elements AR, as well as the open-source tool CellProfiler [19]. Both programs first segment the cell nuclei (Figure 2F), followed by detection of the cell boundaries (Figure 2G,J), whereby not only epithelial cells can be recognized but also fibroblast-like ones (Supplementary Figure S6). Following single-cell and nuclear recognition, segmentation of the N protein (Figure 2H,K) and association of the different masks can be used to distinguish infected from non-infected cells. Due to the mask association (per single-cell), virus particles non-specifically bound to the glass slide can easily be ignored. We provide a detailed walkthrough of the image analysis in our short tutorial (Supplementary Software; sample datasets can be downloaded from https://zenodo.org/records/10417810 (accessed on 7 January 2024)). Using this protocol, we then analyzed cells infected with PUUV and TULV at different time points post-infection (6 h, 12 h, 24 h and 48 h). Interestingly, a positive infection with PUUV, as well as with TULV, could already be detected at 6 h post-infection (Figure 3C), which was not possible using flow cytometry (Supplementary Figure S2). After 48 h, we found similar infection rates as measured by flow cytometry when using the same input virus (Supplementary Figure S7). Over the course of infection, we observed a correlation between the number of infected cells and the intensity of the segmented signal (Figure 3A,B). This observation implies that within the initial 48 h of infection, the two parameters are linked, and the quantification mainly monitors the primary infection. To test this conclusion, we used the supernatant of all time points to infect new Vero E6 cells for a subsequent 48 h period. Interestingly, we observed infection rates at background levels for all supernatant samples except the 48 h time point (Supplementary Figure S8), supporting our conclusion that we are effectively detecting the ongoing primary infection process (Figure 3). Based on these findings, we recommend the 48 h time point post-infection, characterized by the highest signal intensity, as the optimal time frame for quantifying PUUV and TULV stocks.

### 3.3. Custom Analysis Software Tools Allow for the Quick Visualization of Image Data and the Calculation of Infection Rates

**Figure 4:**
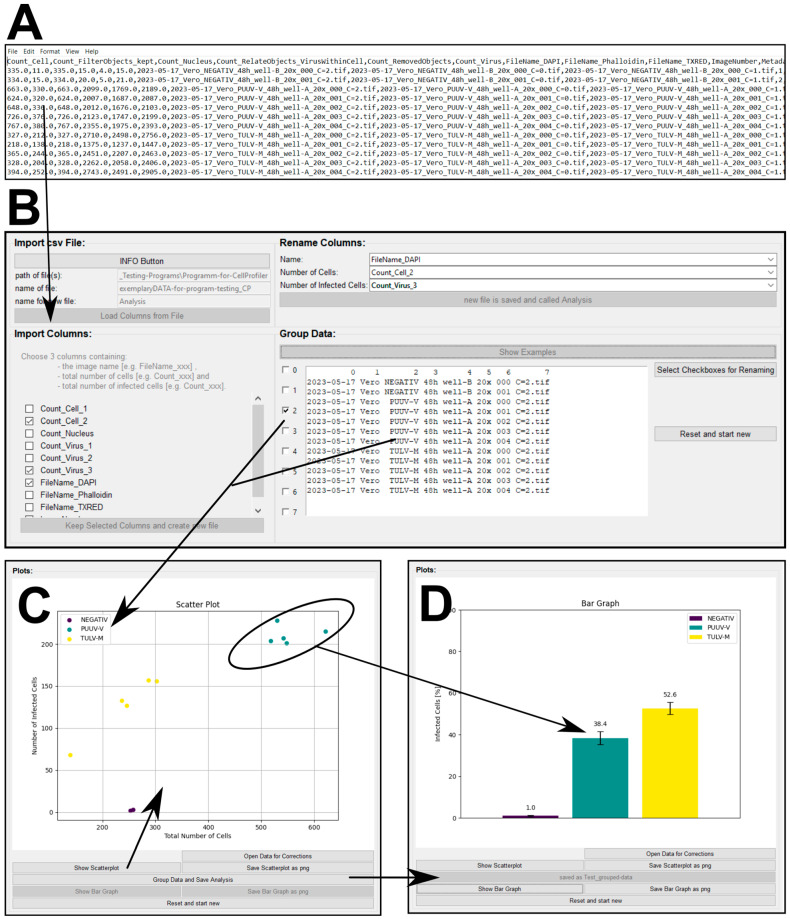
Custom software tool for processing CellProfiler output. (**A**) An exemplary csv file as a result of using CellProfiler to quantify PUUV and/or TULV infection after 48 h. (**B**) Graphical user interface (GUI) of the software that allows for the processing of csv files. The software tool summarizes and groups’ important data columns based on the image name to obtain visual output in the form of scatterplots (**C**) or bar graphs (**D**).

The image analysis pipeline discussed above produces signal intensity datasets in tabular format, which require further processing. To ease this step, we provide two versions of our open-source software equipped with a graphical user interface (Figure 4B–D) so that no programming skills are required. After downloading, the software to process CellProfiler or Nis Elements data is ready to use without further installation. The raw data can be visualized as a scatterplot (Figure 4C), whereby datasets are color-coded and can be further summarized as an average percentage of infected cells (Figure 4D). For more detailed information, please refer to the provided software manual packaged together with the Supplementary Software. Finally, to convert the fraction of infected cells into infectious particles per milliliter, three parameters are necessary: (1) the percentage of infected cells as given by our analysis tool (e.g., 50.9% converted as a fraction (0.509)); (2) the used cell numbers (e.g., 100,000 cells per well); and (3) the amount of virus suspension (e.g., 5 µL per well) used to include the used dilution (1:200) to finally calculate infectious particles per mL.

Fraction of infected cells [decimal] × cell number × dilution [e.g., 5 µL = 1:200]

= infectious particles/Milliliter

Example: 0.509 × 100,000 × 200 = 10,180,000 or 10.18 × 10^6^ infectious particles per mL.

### 3.4. Automated Image Analysis Allows for the Extraction of Structural Parameters to Describe the Infection Status

**Figure 5:**
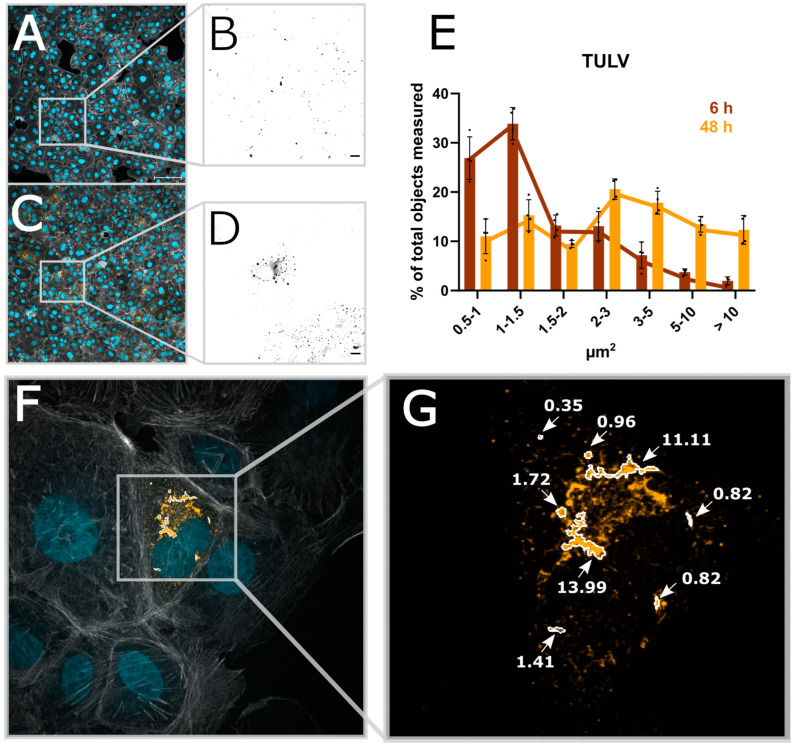
Automated image analysis allows for the extraction of structural parameters. (**A**) A micrograph taken with 20× magnification of Vero E6 cells infected with TULV 6 h post-infection. (**B**) The viral N protein; scale bar = 10 µm. (**C**,**D**) The 48 h post-infection time point of TULV infection. (**E**) The size of TULV-N segmented particles at either 6 h or 48 h post-infection. (**F**) A 100× magnification of 48 hpi TULV-infected Vero E6 cells, (**G**) Exemplary TULV-N structures and the correlating size.

During the analysis of our acquired images, we noticed that the size of the N protein structures per cell changes over the course of infection. This corresponds to different levels of N protein expression per cell, presumably corresponding to different stages of viral replication. We thus wanted to test if such changes could be visualized using our image analysis pipeline. Indeed, in addition to the percentage of infected cells, other parameters, such as the size of the N protein segments, can easily be extracted and displayed over time (Figure 5E and Supplementary Figure S9). Both PUUV-N and TULV-N particles are detected after 6 h as small and mostly round-shaped structures. While the number of particles increases over time, their shape also changes, which can be measured using the area of the segmented N protein signal. TULV-N, as well as forming bigger round particles, also form long fiber-like structures which can then be investigated in detail using higher magnification (Figure 5F,G).

## 4. Conclusions

We developed an optimized workflow to determine the infectious titer of two orthohantavirus strains, PUUV and TULV, using flow cytometry and fluorescence imaging. Our improved workflow allows us to identify infected cells as early as 6 h post-infection, but we recommend using the 48 h time point for the robust recognition of infected cells. We want to point out that in our study, we typically used low MOIs to save material and avoid challenges in obtaining high titers. It would be interesting to determine the titer over a larger range of MOI and compare our methods with, for example, TCID50 curves. In this study, we provide a detailed protocol of the sample preparation procedure and include new user-friendly and open-source software tools to quickly process results from automated image analysis. In conclusion, our microscopic assay enables the fast identification of infected cells with low cell loss and minimized material requirements. Imaging-based quantification furthermore allows for the extraction of structural and positional features of the detected signal, which can later be used for the correlation of other phenotypes such as replication status, as we show, or cell cycle, for example. 

## Data Availability

Supplementary data are provided. Example raw data for testing the provided software are available at https://doi.org/10.5281/zenodo.10417810.

## Supplementary Materials

The following supporting information can be downloaded at: https://www.mdpi.com/article/10.3390/v16020269/s1, Figure S1: Comparison of the infectious titer of different enveloped RNA virus; Figure S2: Flow cytometry measurements of Vero E6 cells 6 h post infection with PUUV and TULV; Figure S3: Percentual decrease of Vero E6 cells due to consecutively washing steps in PBS during the flow cytometry staining protocol; Figure S4: Staining background created by different usage of antibodies (AB). Figure S5: Exemplary gating settings for flow cytometric analysis of infected cells; Figure S6: Software recognition of infected cells with a fibroblastic cell-type; Figure S7: Quantification of PUUV and TULV infectious titer using microscopy or flow cytometry; Figure S8: Release of infectious viruses after different time points; Figure S9: Size distribution of identified segments of PUUV-N identified in infected cells at different time points (6 h vs. 48 h) in Vero E6 cells.
